# Health Perception and Anxiety Among Internally Displaced and Non-Displaced Israeli Adults: The Mediating Role of Emotional Well-Being and Functioning

**DOI:** 10.3390/healthcare13222994

**Published:** 2025-11-20

**Authors:** Orly Toren, Tziporah Novoseller, Dahlya Selig, Mayan Bar On, Galit Madar

**Affiliations:** Faculty of Healthcare Sciences, School of Nursing, Ono Academic College, Academic Boulevard, Kiryat Ono 5545179, Israel

**Keywords:** anxiety, mediating model, displaced persons

## Abstract

**Background**: Forced displacement is a significant public health challenge associated with deteriorated mental and physical health outcomes. Following the outbreak of Israel’s ‘Iron Swords’ war on 7 October 2023, more than 250,000 citizens were evacuated from their homes. Previous research has consistently documented elevated anxiety and poor health perception among displaced populations; however, the extent to which displacement itself contributes to anxiety has not been directly examined or established. **Objective**: The objective was to compare levels of anxiety and health perception between internally displaced and non-displaced Israeli adults and examine the mediating roles of emotional well-being and emotional functioning within the framework of Hobfoll’s Conservation of Resources (COR) theory. **Methods**: A cross-sectional, comparative quantitative study was conducted using validated self-report questionnaires to assess health perception (SF-36) and anxiety (GAD-7). The study sample comprised 98 adults, including 46 displaced individuals and 52 participants from the general population. Differences in health dimensions and anxiety levels were analyzed using *t*-tests, correlation analyses, and regression models. To advance understanding beyond previous research, mediation analysis based on the Conservation of Resources (COR) theory was employed, enabling identification of the psychological mechanisms through which displacement influences anxiety. **Results**: Displaced participants reported significantly lower scores across all SF-36 dimensions and significantly higher anxiety levels compared to the general population. Regression analyses indicated that emotional well-being and emotional functioning were significant predictors of anxiety, whereas displacement status alone was not a direct predictor once mediators were included. Mediation analysis further demonstrated that both emotional well-being and emotional functioning fully mediated the relationship between displacement and anxiety **Conclusions**: Forced displacement has lasting negative effects on mental health, primarily through the erosion of emotional and functional resources. These findings highlight the importance of interventions aimed at strengthening psychological resilience and continuity of care. Study limitations include a cross-sectional design, reliance on self-reported data, and relatively small sample, which may limit generalizability.

## 1. Introduction

Internal displacement, as defined by the United Nations Guiding Principles, refers to situations in which individuals or groups are forced to flee their homes due to conflict, violence, persecution, or disasters. Unlike refugees, internally displaced people (IDPs) remain within the borders of their own country [1].

As of 2021, there were approximately 48 million people worldwide who had fled or were forced to leave their homes in areas affected by conflict or war, relocating to other parts of their own countries [2]. The largest internally IDPs since World War II has been observed in Ukraine, with 6.5 million IDPs and 6.3 million refugees [3].

Internal displacement is closely linked to widespread impacts on both the mental and physical health of affected populations. Recent studies indicate a high prevalence of medical problems among IDPs, including respiratory infections, chronic pain, sleep disorders, and reduced functioning, alongside elevated rates of anxiety, depression, and Post Traumatic Stress Disorder. A recent study found that approximately 45% of IDPs reported poor physical health, 14% reported anxiety, and 18% reported depression [4].

In Israel, the “Iron Swords” war, which erupted on 7 October 2023, led to the evacuation of hundreds of thousands of Israeli citizens from their homes. According to data collected by the Knesset Research and Information Center, approximately 253,000 Israelis were forced to flee and evacuate their homes in the first days following the surprise attack carried out by the Hamas terrorist organization on communities near the Gaza border on 7 October 2023 [5]. As of September 2024, about 143,000 citizens still remain displaced from their homes [6].

This situation poses a unique challenge for Israel’s healthcare system due to its physical and mental health implications. Various communities from both the north and the south were evacuated to hotels across the country, where they resided for many months. In some of these communities, there are signs of social disconnection and weakening community ties [6], a reality that further exacerbates the health-related challenges—both physical and mental.

**Anxiety**—caused by displacement and long periods in temporary housing, which is often marked by overcrowding, lack of privacy, and limited educational or employment opportunities—place IDPs at high risk of anxiety, depression, loneliness, and other mental health problems. Numerous studies report a high prevalence of mental disorders, particularly anxiety, among IDPs. Key risk factors identified include heightened exposure to trauma and increased psychological distress in the aftermath of displacement [7,8].

A longitudinal study conducted in Israel after 7 October found that forced displacement had a strong and lasting impact on anxiety levels, with elevated GAD-7 scores persisting for at least 90 days [9]. Five months after the outbreak of war, 32% of participants exceeded the moderate anxiety threshold (GAD-7 ≥ 10), compared with 24% three months later, indicating enduring anxiety symptoms. Overall, 75% of participants reported at least one symptom of anxiety, depression, or PTSD on day 1, a rate that slightly declined to 69% on day 30 and to 67% on day 90.

**Similarly**, Ulke et al. [8] showed that the psychological effects of displacement can persist for decades: in Germany, IDPs reported significantly higher anxiety rates than non-displaced peers, even in old age. Women were particularly vulnerable, underscoring the importance of targeted support for high-risk groups.

A recent systematic review and meta-analysis based on WHO estimates reported that the prevalence of mental disorders among individuals exposed to conflict is approximately **22%**, including anxiety disorders. In comparison, the rate of anxiety among internally displaced persons (IDPs) in Mogadishu was found to be 43.7% [10,11]. Elevated anxiety levels persisted many years after displacement, even among individuals who had resettled and achieved relative stability [12]. In comparison, the last national survey in Israel estimated the lifetime prevalence of anxiety disorders at 5.2% [13]. These figures highlight the disproportionate burden of anxiety among IDPs and the need to explore the mechanisms that underlie this relationship.

**Regarding perceived health**, morbidity and mortality among IDPs are consistently higher than national averages [2]. Displacement, regardless of its cause, often exposes evacuees to harsh physical conditions, limited access to medical services, and difficulties in adhering to treatment regimens. These conditions increase vulnerability to infectious diseases, untreated malnutrition, and chronic illnesses, which may cause health to further deteriorate due to difficulties in adhering to prescribed treatment regimens [14].

Recent evidence highlights that evacuation itself can have adverse health consequences. For example, Hua et al. (2024) found that evacuation of assisted living residents during Hurricane Irma was associated with increased rates of emergency department visits and hospital admissions compared to those who sheltered in place [14].

Following the Fukushima disaster, evacuees experienced increased chronic disease rates and mortality, illustrating the long-term health impact of displacement [15,16]. These findings underscore the critical importance of ensuring continuity of care and access to medications in disaster preparedness and response for displaced populations.

Health is a multidimensional construct encompassing biological, psychological, and social dimensions. Self-perceived health is a widely used and reliable indicator of overall well-being, strongly associated with both morbidity and mortality [17]. It reflects individuals’ integrated assessment of their physical, psychological, and social health beyond objective medical indicators [14]. The advantages of self-rated health lie in its ability to capture multiple domains of well-being and to integrate these with short-term changes that contribute to an overall appraisal of health status.

The SF-36 questionnaire [18] is one of the most widely used instruments for assessing perceived health. It is a valid measure developed through extensive cross-cultural and multi-national research. It captures both physical and mental dimensions and in the general population; scores demonstrate a strong correlation between self-rated health and objective medical indicators [19].

War and the forced displacement of populations create a volatile situation that significantly increases the risk of mental health disorders, representing one of the most critical threats to individual well-being [20]. Such mental health problems are particularly prevalent among IDPs compared with the general public [21,22].

Studies show that the SF-36 effectively differentiates between displaced and non-displaced populations, highlighting significant gaps in mental and physical health. IDPs generally report lower mental health scores—associated with trauma, anxiety, and depression—and reduced physical functioning linked to limited healthcare access [4,19]. Its strong reliability makes it a valuable instrument for assessing perceived health and tracking health changes over time.

**Theoretical Model**. This study is based on Hobfoll’s Conservation of Resources (COR) model [23,24]. The model was developed to explain how the loss of, or threat to, personal, social and material resources leads to stress and psychological responses such as anxiety and depression. According to this model, stress arises from the loss or threat to personal resources (e.g., health, self-esteem), social resources (e.g., family support), and material resources (e.g., home, livelihood). Among IDPs, such resource depletion undermines emotional regulation and resilience, increasing anxiety and impairing health. Integrating the assessment of physical and mental health through instruments like the SF-36 enables examination of how resource loss mediates the relationship between traumatic exposure and anxiety levels [23]. 

**Figure 1:**
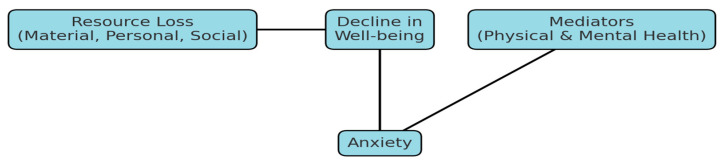
The COR Model for Predicting Anxiety.

Growing evidence suggests that emotion regulation difficulties serve as a central psychological mechanism linking traumatic exposure and adverse post-displacement conditions to anxiety among IDPs. The loss of personal and social resources following displacement undermines emotion regulation capacities, thereby elevating the risk for anxiety and depression [4]. Consistent with this, a systematic review reported that maladaptive strategies—particularly expressive suppression—are strongly related to increased anxiety and psychological distress in refugee populations [20]. Comparable findings in trauma-exposed samples confirm that emotion dysregulation functions as a direct mediator between trauma and internalizing symptoms such as anxiety and depression, Figure 1 [25,26].

## 2. Purpose

The purpose of this study is to examine the impact of displacement on mental and physical health by comparing levels of anxiety, perceived health, and mental well-being between displaced persons and the general population in Israel, and to assess the role of health (both physical and mental) as mediating factors in this relationship.

Accordingly, the study investigated whether differences exist in anxiety levels and perceived health between the displaced population in Israel and the general population, and whether perceived physical health, mental well-being, and emotional functioning mediate the relationship between displacement status and anxiety levels.

## 3. Method

A comparative quantitative cross-sectional study was conducted using self-report questionnaires. The study included 98 men and women over the age of 18, comprising (1) evacuees from the south or northern parts of Israel who had not returned to their homes at the time of data collection and (2) individuals from the general population who had not been evacuated from their homes. To maintain a similar proportion between the two groups, approximately half of the study participants who completed the questionnaire were sampled from the general population (52 individuals, representing 53.1% of all respondents), and the other half from the IDPs (46 individuals, representing 46.9% of all respondents). Inclusion criteria encompassed all adults in Israel who can read and write in Hebrew, Table 1.

The majority of respondents were women and held an academic degree. The mean age in the IDP group was 38.33 years (SD = 14.93), compared to 35.75 years (SD = 15.41) in the general population group, with no statistically significant difference between the two. Likewise, no significant differences were observed between the groups in the other demographic variables, including marital status, level of religiosity, and economic status.

## 4. Research Tools

**Part A**—Perceived Health: This was measured using the SF-36 questionnaire [18]. The questionnaire is designed for self-administration among both healthy and ill populations, as it is sensitive to deterioration in health status. It is suitable for all ages and contexts and has been validated in diverse cultural populations. The SF-36 was developed by the RAND Corporation, Santa Monica, CA, USA. A commercial version is available via QualityMetric Incorporated, Johnston, Rhode Island, USA. The SF-36 consists of 36 items measuring eight health domains. The items are multiple-choice, and the response scales vary depending on the question. Higher scores indicate higher self-perceived health.

The SF-36 has demonstrated strong reliability across diverse populations. Cronbach’s α values typically range from 0.76 to 0.95 across subscales, with overall scores around 0.87 [27,28,29]. Test–retest reliability is also high, for both physical and mental component summaries [28]. These findings affirm the SF-36’s robust psychometric performance across different demographic and clinical groups. Given the complexity of its scoring and dimension construction, analyses were performed in accordance with the procedures described in the original paper [18].

The health domains assessed and the reliability coefficients for each subscale are presented below. For each subscale, a total sum score of the responses was calculated.

### 4.1. Dimensions Related to the Physical Health

Physical Functioning: 10 items assessing limitations in daily activities due to health status. Example: *“To what extent does your health limit you in lifting or carrying groceries?”* Reliability (Cronbach’s Alpha) = 0.95.

Role Limitations due to Physical Health: 4 items assessing the impact of physical limitations on work or daily activities during the past month. Example: *“During the past month, have you cut down the amount of time you spent on work or other activities?”* Reliability (Cronbach’s Alpha) = 0.85.

Bodily Pain: 2 items assessing the respondent’s physical functioning in relation to pain. Example: *“How much bodily pain have you had during the past four weeks?”* Reliability (Cronbach’s Alpha) = 0.91.

General Health: 5 items assessing the respondent’s overall physical functioning. Example: *“In general, would you say your health is …?”* Reliability (Cronbach’s Alpha) = 0.85.

### 4.2. Dimensions Related to the Emotional Health

Role Limitations due to Emotional Problems: 3 items assessing mental functioning over the past month. Example: *“During the past month, have you accomplished less than you would like as a result of any emotional problems?”* Reliability (Cronbach’s Alpha) = 0.87.

Energy/Fatigue: 4 items assessing feelings and how things have been going during the past month. Example: *“Did you feel full of pep?”* Reliability (Cronbach’s Alpha) = 0.92.

Emotional Well-Being: 5 items assessing feelings and general mental state over the past month. Example: *“Have you felt so down in the dumps that nothing could cheer you up?”* Reliability (Cronbach’s Alpha) = 0.88.

Social Functioning: 2 items assessing the extent to which physical or emotional health has interfered with normal social activities during the past month. Example: *“During the past four weeks, to what extent has your physical health or emotional problems interfered with your normal social activities (like visiting with friends, relatives etc.?”* Reliability (Cronbach’s Alpha) = 0.84.

**Part B**—Generalized Anxiety Perception: Generalized anxiety was measured using the self-report GAD-7 questionnaire [30] which was developed by Spitzer, Kroenke, Williams et al. in the USA and is available for reproduction without charge as of 2010. The questionnaire consists of seven items describing symptoms of generalized anxiety, rated on a four-point Likert scale (0—*not at all*; 3—*nearly every day*). Example: *“over the last two weeks how often have you been bothered by feeling nervous, anxious, or on the edge*”. Higher scores indicate higher levels of anxiety. The GAD-7 is widely used for both research and clinical purposes to screen for anxiety symptoms in the general population and in clinical populations. It has strong psychometric properties, including high internal consistency (Cronbach’s alpha = 0.89) [30,31,32] and high validity indices [27,28]. In the present study, the questionnaire demonstrated excellent reliability (Cronbach’s alpha = 0.96). The total anxiety score was derived by summing the responses to the seven GAD-7 items, each rated on a 4-point scale from 0 (“not at all”) to 3 (“nearly every day”). Thus, the total score ranges from 0 to 21, with higher values reflecting greater anxiety severity.

In accordance with standard scoring conventions, anxiety levels were classified as follows:

- 0–4: minimal anxiety;
- 5–9: mild anxiety;
- 10–14: moderate anxiety;
- 15–21: severe anxiety.

**Part C**—Demographic Questionnaire including personal details such as gender, age, country of birth, education, etc.

## 5. Sampling

The survey was distributed online between 13 October and 18 November 2024, approximately one year after the displacement event. A convenience sample combined with the snowball sampling method was used. Participants completed anonymous self-report questionnaires via Google Forms. The survey link was initially distributed by the research team through targeted WhatsApp and Facebook groups that included IDPs groups and general population groups (e.g., community and professional networks). They were asked to forward the online link to additional acquaintances for completion. The questionnaires were completed online by the study participants, accessed either through their mobile phones or personal computers.

## 6. Statistical Analysis

Descriptive statistics were employed to summarize the participants’ sociodemographic characteristics, including the distribution of key research variables both overall and across relevant background factors (e.g., sex, age, education). Additionally, correlations between various variables were calculated.

Internal reliability the SF-36 subscales and for the Gad-7 questionnaire was assessed using Cronbach’s alpha. Differences between the groups were examined using *t*-tests for independent groups, and multivariate regression analyses examining the combined effect of all predictors on anxiety. A mediation model [30,31] was tested to estimate the total and indirect effects of the emotional variables on anxiety levels, specifically assessing their contribution to explaining the relationship between IDPs status and anxiety.

## 7. Ethical Considerations

Ethical approval was obtained from the Ethics Committee of the Research Authority at Ono Academic College. In Israel, the regulations for human subject research distinguish between interventional studies and studies based on existing data and questionnaires. This type of research is defined, among other things, as ‘A prospective study in which health data is collected from individuals through direct contact with them.’ Such studies do not require signed informed consent (https://www.gov.il/blobFolder/guide/protocol-of-medical-research-involving-human-subjects/he/clinical_trials_cth_GLOSSARY.pdf accessed on 9 October 2025).

Since this study is survey-based rather than interventional, the research questionnaire provided participants with a clear explanation of the study and the significance of their participation. Each participant had the option to decline participation or withdraw at any time. To safeguard participant rights, all participants were informed about the study details, including the estimated time required to complete the questionnaires. Furthermore, all collected data remained entirely anonymous and confidential. Completion of the questionnaire was considered as consent to participate.

## 8. Results

Table 2 shows that across all eight dimensions assessing physical and mental health, the IDPs had significantly lower scores than the general population. In contrast, the IDPs showed significantly higher anxiety scores.

Table 3 presents significant differences between the IDPs and the general population, with IDPs showing a substantially higher proportion of high anxiety scores (χ [2](1) = 27.7, *p* < 0.001). Specifically, 36.7% of the displaced group reported high anxiety levels (total score > 11), compared to 13.3% of the general population.

Table 4 indicates that among IDPs, all dimensions of perceived health were significantly and negatively correlated with anxiety, except for physical role limitations. A similar pattern was found in the general population, though no correlation was observed between physical functioning and anxiety.

In both groups, strong negative correlations were evident between role limitations due to emotional problems, emotional well-being, and social functioning, and anxiety. In other words, lower emotional role functioning, poorer emotional well-being, and reduced social functioning were all associated with

To predict the dependent variable, anxiety, a multiple linear regression analysis was performed, incorporating all study variables along with group affiliation (IDPs vs. general population) (Table 5). The variable bodily pain was not included in the regression analysis due to multicollinearity (VIF = 3.2) with other physical health indicators and lack of statistical significance; it was excluded in order to maintain model parsimony and prevent artificial inflation of the explained variance. The variable social functioning was omitted as it did not align with the theoretical framework underlying the study.

Prior to regression analysis, all assumptions were tested and met. Relationships between variables were linear, residuals were approximately normally distributed and homoscedastic, errors were independent (Durbin–Watson = 2.16), no multicollinearity was detected (VIFs < 2.5), and no influential outliers were identified (Cook’s D < 1.0; Leverage < 0.35).

Preliminary analyses showed that the group variable (general population vs. IDPs) explained 34% of the variance in anxiety levels. However, when perceived health variables were added to the model (Table 5), the effect of the group became non-significant. In contrast, two variables—emotional role functioning (β = 0.351, *p* < 0.01) and emotional well-being (β = 0.36, *p* < 0.01)—were significant predictors of anxiety levels. The expanded model was statistically significant [F(7, 89) = 33.82, *p* < 0.01], accounting for 72.7% of the variance in anxiety.

**Figure 2:**
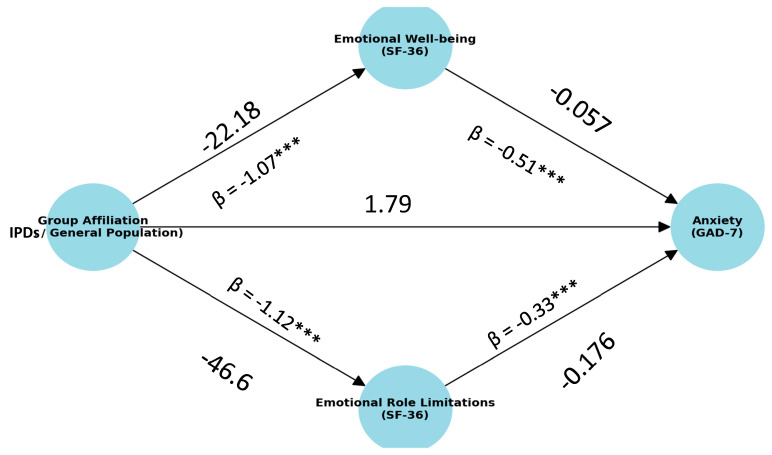
Mediation Model to predict anxiety. Note: Paths marked with β indicate standardized regression coefficients. *p* < 0.001 ***.

Given these findings, and the identification of emotional well-being and emotional role functioning as significant predictors, a mediation model [33,34,35,36] was tested to assess the total and indirect effects of these variables on anxiety, with particular focus on their role in explaining the relationship between evacuation status and anxiety. The mediation model was estimated using OLS regression with bias-corrected bootstrapping (5000 resamples) via the PROCESS macro for SPSS (Model 4) [33,34,35,36]. Note that the ellipses in Figure 2 were used solely for graphical simplicity and do not represent latent variables.

Although the sample size was relatively small (N = 98), prior simulations indicate that samples of 70–100 participants provide adequate power (~0.80) to detect medium indirect effects [35]. The potential limitations of the small sample size—such as wider confidence intervals and reduced generalizability—are now acknowledged and discussed in the revised manuscript.

As presented in Figure 2, Regression and mediation analyses revealed that the IDPs scored significantly lower than the general population in Emotional Well-being (t (96) = 6.21, *p* < 0.001, β = −1.07; MΔ = −22.18) and Emotional Role Limitations (t (96) = 6.70, *p* < 0.001, β = −1.12; MΔ = −46.6). Both variables were strong negative predictors of anxiety, with Emotional Well-being showing a large effect (t (94) = 7.12, *p* < 0.001, β = −0.517) and Emotional Role Limitations a moderate effect (t (94) = 4.45, *p* < 0.001, β = −0.331). Initially, group affiliation predicted an 8.34-point higher anxiety score among the IDPs (t (96) = 7.156, *p* < 0.001), but this effect became non-significant (1.79 points) after including the mediators. Bootstrap analyses confirmed significant indirect effects for both Emotional Well-being (3.91 points, 95% CI [2.49, 5.50]) and Emotional Role Limitations (2.64 points, 95% CI [1.26, 4.28]). Together, these mediators accounted for 6.55 points of the group–anxiety relationship, indicating that nearly all of the difference in anxiety levels between groups was explained by reduced Emotional Well-being and Emotional Role Limitations due to Emotional Problems among the displace.

This finding suggests that the difference in anxiety scores between the IDPs and the general population is almost entirely accounted for by the two mediators, both reflecting aspects of emotional status.

## 9. Discussion

Forced displacement has emerged as a major public health challenge with long-lasting implications for mental and physical well-being [1]. Although numerous studies have described the adverse outcomes of displacement, few have explored the underlying mechanisms that explain how these effects occur.

The current study introduces an important and innovative perspective by applying a mediation model grounded in the Conservation of Resources (COR) theory, enabling examination of the psychological pathways through which displacement influences anxiety. This approach extends beyond descriptive comparisons and contributes to a deeper understanding of how emotional well-being and emotional functioning mediate the relationship between displacement and mental health outcomes.

This study provides new evidence on the health consequences of internal displacement in Israel, demonstrating significantly poorer perceived health and higher anxiety among IDPs compared with non-displaced individuals. Beyond confirming known associations, the results reveal the specific mechanisms through which displacement affects mental health, emphasizing the mediating role of emotional well-being and emotional functioning.

The results expand on prior research by demonstrating that even within one year of displacement, emotional well-being and functioning—not displacement itself—were the strongest predictors of anxiety. This highlights the importance of targeting emotional resilience and coping capacities in clinical and community interventions for IDPs.

One year after the war, over a third (36%) of evacuees reported high anxiety levels, markedly higher than both the general population and rates reported nine months after displacement [9]. Taken together, IDPs should be recognized as a high-risk group requiring targeted clinical interventions to alleviate anxiety, enhance emotional resources, and prevent the escalation of psychological distress.

Among IDPs, stress and depletion of personal resources were found to predict heightened anxiety [37]. Longitudinal evidence from Israeli war veterans showed that loneliness and poor subjective physical health reinforce each other over time, with physical health problems both reflecting and aggravating symptoms of PTSD and anxiety [38]. More recently, the health and environmental consequences of the Gaza conflict highlighted the profound physical and psychological toll of prolonged exposure to insecurity and instability, reinforcing the notion that deteriorated physical health and weakened resilience serve as significant predictors of long-term psychological distress [39]. Collectively, these studies strengthen the interpretation that poor perceived health is not only a subjective reflection of adversity but also a key marker of depleted resilience and social resources, underscoring the need for comprehensive interventions to mitigate long-term mental health.

A key and innovative finding of this study is the identification of emotional well-being and emotional role limitation as mediators in the relationship between displacement and anxiety. While most studies have conceptualized evacuation as a direct cause of heightened anxiety [37,38,39] and longitudinal evidence from Israel following 7 October demonstrated persistently elevated anxiety levels among evacuees months after displacement [9], the present findings suggest that this effect is not direct but operates primarily through these mediating factors. In fact, anxiety is explained almost entirely through the erosion of psychological resources—diminished sense of control, weakened social belonging, and impaired emotional coping capacity. This mediation approach provides new theoretical and empirical insights into the psychological mechanisms underlying anxiety among IDPs—an area rarely examined in Israeli or international research. The findings are consistent with Hobfoll’s Conservation of Resources theory, which highlights the role of resource loss in psychological distress and supports the model’s relevance to the context of forced displacement. Moreover, although all participants were exposed to the same national environment of war and insecurity, the IDP group faced an additional layer of stress stemming from forced relocation, prolonged uncertainty, and disruption of community ties. These circumstances suggest that the elevated anxiety observed in this group is primarily associated with the displacement experience itself, rather than with general wartime exposure.

In contrast to prior studies reporting greater psychological vulnerability among women after forced evacuation [8], gender was not a significant predictor of anxiety in the present study. This may reflect sample characteristics or the context of ongoing war, where men and women were similarly exposed to negative experiences. Nevertheless, examining gender differences remains essential to ensure that interventions are tailored to diverse needs.

Other explanations may be considered as alternatives to our findings. However, in the present study, no significant differences were found between IDPs and the general population regarding emotional well-being or anxiety. One possible explanation is that both groups were exposed to similar contextual stressors, such as ongoing war conditions, socioeconomic disruption, or shared community stress. Moreover, data collection was conducted over a short and identical time period for both groups, minimizing the influence of temporal or situational variation. These factors may have reduced the likelihood of observing displacement-specific effects. This interpretation suggests that the broader crisis context may have affected both groups similarly, underscoring the pervasive impact of collective adversity on psychological functioning.

Study limitations and future research directions are discussed in the following section. Still, the consistency and robustness of the findings point to clear trends that are supported by theoretical models and previous research.

## 10. Conclusions and Final Considerations

This study underscores the substantial impact of forced internal displacement on both perceived health and anxiety among Israeli adults following the “Swords of Iron War.” By identifying emotional well-being and emotional functioning as mediators in this relationship, the findings highlight the critical role of psychological resources in shaping individuals’ ability to cope with trauma and instability. These results deepen our understanding of how displacement influences mental health through emotional mechanisms rather than through displacement alone.

However, although the study met the required statistical power to detect medium-sized effects, the findings should be interpreted with caution given the cross-sectional design and modest sample size. The study provides preliminary insights that may inform future clinical and policy considerations; however, further research using longitudinal and representative samples is needed before drawing firm conclusions or making direct recommendations for practice or policy.

## 11. Limitations

Despite its valuable insights, this study has several methodological limitations that should be considered when interpreting the results. First, the cross-sectional design precludes any inference of causality, as it captures associations at a single point in time.

Second, the use of self-report questionnaires may have introduced response bias, as participants’ perceptions and willingness to report distress could vary depending on individual factors. Third, the use of a relatively small convenience sample may have constrained the statistical strength of the findings and limit the applicability to the broader displaced population (external validity and generalizability). Nevertheless, the consistency of the findings across multiple indicators of emotional and physical health supports the robustness of the observed relationships.

Participation in the online survey may have been influenced by respondents’ emotional state and motivation to engage. While this factor could theoretically affect participation rates, the relatively high levels of anxiety and distress observed in this study suggest that individuals experiencing significant psychological strain were well represented. It is therefore likely that the inclusion of additional highly distressed individuals would have further reinforced the observed trends rather than altered them.

Future research should employ larger, randomly selected, and longitudinal samples, as well as qualitative or mixed-method approaches, to validate and expand upon the current results.

## 12. Implications for Public Health, Policy, and Society

The findings have significant implications for public health and governmental policy. Comprehensive strategies should ensure continuity of care, strengthen emotional resilience, and promote sustainable psychological recovery among IDPs. Psychological support must be an integral component of displacement response plans, emphasizing the enhancement of emotional functioning, coping capacity, and overall well-being. For example, structured emotional resilience programs implemented within temporary housing settings could help displaced adults develop more effective emotion regulation and stress management skills. Additionally, integrating brief group-based interventions—such as mindfulness training, cognitive-behavioral coping workshops, or psychoeducational sessions—into community clinics and temporary accommodation facilities for IDPs could further reduce anxiety symptoms and promote emotional well-being.

To implement these goals effectively, interdisciplinary collaboration between healthcare providers, mental health professionals, social services, and community organizations is essential. Such cooperation can facilitate coordinated intervention programs, improve access to care, and ensure that IDPs receive integrated medical and psychosocial support. Health authorities should also invest in training for professionals working with IDPs to recognize emotional distress early and strengthen adaptive coping mechanisms.

From a broader societal perspective, policies should focus on rebuilding community networks and fostering social cohesion, which serve as protective factors against anxiety and long-term psychological distress. Strengthening these community ties, alongside evidence-based resilience programs, can enhance recovery and promote a more holistic public health response in future displacement crises.

## 13. Future Research Directions

Future research should examine longitudinal trajectories of health and anxiety among IDPs and explore gender, age, and socioeconomic differences in emotional adaptation. Qualitative studies would also provide deeper insight into the lived experiences of IDPs, complementing quantitative evidence.

In conclusion, while this study provides important preliminary insights into the relationship between displacement, emotional functioning, and anxiety, the cross-sectional design and modest sample size limit the extent to which causal or long-term conclusions can be drawn. Accordingly, the findings should be interpreted as indicative rather than conclusive. The implications for clinical practice and public health policy are therefore presented as potential directions for future intervention and planning rather than as definitive recommendations. Further longitudinal and intervention-based research is needed to verify these associations and to provide stronger evidence for developing effective clinical and public health responses for IDPs.

## Figures and Tables

**Table 1:** Socio-demographic characteristics of the IDPs compared to the general population.

(*n* = 52) General Population					(*n* = 46) IDP			
Significance	%	*N*	Categories	Variable	%	*N*	Categories	Variable
N. S	38.5	20	Male	**Gender**	30.4	14	Male	**Gender**
	61.5	32	Female		69.9	32	Female	
N. S	17.3	9	High school	**Education**	19.6	9	High school	**Education**
	21.2	11	Vocational		19.6	9	Vocational	
	61.5	32	Academic		60.9	28	Academic	
		**S.D.**	**Mean**			**S.D.**	**Mean**	
N. S		1.35	35.75	**Age**		1.15	38.33	**Age**
			(21–74 range)				(19–81 range)	
N. S		1.68	1.35	**No. of children**		1.46	1.15	**No. of children**
			(0–7)				(0–4 range)	

**Table 2:** Comparison of health measures and anxiety between the IDPs and the general population.

Health Categories		N	Mean	SD	t Scores
+ Physical Functioning	IDPs	46	74.56	29.18	t (76.21) = 2.76 ***
	General Population	52	88.55	19.20	
Physical Role Limitation	IDPs	46	38.04	41.075	t (96) = 3.86 ***
	General Population	52	68.26	36.41	
+ Emotional Role Limitation	IDPs	46	7.24	20.97	t (76.05) = 6.69 **
	General Population	52	53.84	42.85	
Energy/Fatigue	IDPs	46	28.36	14.94	t (96) = 6.18 ***
	General Population	52	48.17	16.86	
Emotional Well-Being	IDPs	46	33.73	18.86	t (96) = 6.20 ***
	General Population	52	55.92	16.51	
Social Functioning	IDPs	46	39.40	23.85	t (96) = 4.68 ***
	General Population	52	60.81	21.44	
Bodily Pain	IDPs	46	66.03	25.09	t (96) = 4.33 ***
	General Population	52	85.28	18.70	
General Health	IDPs	46	53.47	24.33	t (96) = 2.83 ***
	General Population	52	66.25	20.26	
Anxiety	IDPs	46	14.93	6.08	t (96) = 7.15 ***
	General Population	52	6.59	5.44	

*p* < 0.001 ***, *p* < 0.01 **. Note. For each variable, the type of *t*-test was determined based on Levene’s test for equality of equal variances not assumed); all other variables were analyzed using the homoscedastic *t*-test (equal variances assumed). Variables marked with a plus sign (+) were analyzed using the heteroscedastic *t*-test (variances).

**Table 3:** Differences between the groups in the prevalence of moderate and high anxiety levels.

	IDPs		General Population		
Anxiety level	N (=46)	%	N (=52)	%	χ2
Up to score 10	10	10.2	39	39.8	χ2(1) = 27.7, *p* < 0.001
Above score 11	36	36.7	13	13.3	

**Table 4:** Pearson correlations between health perception and Anxiety among IDPs and general population.

	Physical Functioning	Physical Role Limitation	Emotional Role Limitation	Energy/Fatigue	Emotional Well-Being	Social Functioning	Bodily Pain	General Health
IDPs (N = 46)								
Anxiety	−0.299 *	−0.287	−0.646 **	−0.576 **	−0.759 **	−0.661 **	−0.418 **	−0.416 **
General Population (N = 52)								
Anxiety	0.002	−0.568 **	−0.633 **	−0.569 **	−0.632 **	−0.602 **	−0.302 *	−0.391 **

*p* < 0.05 *, *p* < 0.01 **.

**Table 5:** Multiple regression analysis for predicting anxiety.

Model	B	Std. Error	Beta	t
(Constant)	14.09	2.35		10.66
Group (IDPs = 1)	1.348	1.047	0.095	1.288
Physical Functioning	0.008	0.022	0.028	0.383
Physical Role Limitation	−0.026	0.013	−0.149	−1.914
Emotional Role Limitation	−0.060	0.014	−0.351	−4.380 **
Energy/Fatigue	−0.064	0.03	−0.17	1.95
Emotional Well-Being	−0.202	0.042	−0.360	−4.769 **
General Health	0.012	0.027	0.038	0.445

*p* < 0.01 **.

## Data Availability

The datasets used and analyzed during the current study are available from the corresponding author upon reasonable request. Data is stored at the statistician (GM) personal computer.

## Abbreviation

IDPs	Internally displaced persons
